# Models of respiratory disease symposium

**DOI:** 10.1186/1476-9255-10-S1-I1

**Published:** 2013-08-14

**Authors:** Kathy H Abbott-Banner, Anthony Holmes, Ian Adcock, Navin L Rao, Edward Barrett, Richard Knowles

**Affiliations:** 1 Present address: Verona Pharma plc, Suite 21 - Alpha House, 100 Borough High Street, London, SE1 1LB, UK; 2 NC3Rs, Gibbs Building, 215 Euston Road, London, NW1 2BE, UK; 3 National Heart and Lung Institute, Imperial College London, Dovehouse Street, London, SW3 6LY, UK; 4 Janssen Research and Development LLC, Immunology, San Diego, CA 92121, USA; 5 Lovelace Respiratory Research Institute, USA; 6 Arachos Pharma Ltd, The Incubator, Stevenage Bioscience Catalyst, Gunnels Wood Road, Stevenage, SG1 2FX, UK

## Introduction

The symposium brought together representatives from the pharmaceutical industry and academia who are actively involved with establishing animal models and also *in vitro* translational assays of respiratory disease. It was the second of its kind, (the first one was held at the Glaxo SmithKline (GSK) Stevenage, UK site in April 2009) exchanging information on the difficult challenge of establishing predictive animal models of respiratory diseases. The organising group, which was led by Dr Kathy Abbott Banner and Prof. Richard Knowles, were originally drawn from the respiratory representatives of the European Federation of Pharmaceutical Industries and Associations (EFPIA) group of companies involved in a bid for EU funds to support work on severe asthma, but have spread the network to include many companies outside this group.

The objectives of the symposium were to share knowledge in an open and collaborative atmosphere, and subsequently reach a consensus on best practice for animal models of respiratory disease. It is hoped that this will lead to decreased unnecessary duplication of animal studies, and thus a reduction in animal numbers.

The symposium was held on September 6^th^ and 7^th^ 2012 at Novartis site in Horsham, UK. There were ~120 participants from 16 different pharmaceutical companies/contract research organisations and 10 academic institutions based in Europe, U.S.A and Australia. The NC3Rs and UBIOPRED were also represented. Key opinion leaders (Prof. Stephen Holgate, Prof. Dave Singh, Prof. Sebastian Johnston and Dr Paul Mercer) gave plenary lectures. In addition there were oral and poster presentations on COPD (**P25-P31**), asthma (**P1-18, P40**), exacerbations (**P19-P24, P41**), fibrosis (**P38, P39**) and pulmonary arterial hypertension (**P37**) and workshops on asthma, COPD and exacerbations. In addition there were posters on inhaled delivery systems (**P35, P36**). As the majority of the abstracts received were on asthma, COPD and exacerbations, this meeting summary will focus on the highlights from those. Several pharmaceutical companies provided sponsorship of the symposium.

## Applying the 3Rs to support respiratory disease research

Asthma is an area of considerable unmet medical need. Few new drugs have made it to the clinic in the last 50 years, with many drugs which perform well in preclinical animal models of asthma, failing in humans. The failure to translate promising drug candidates from animal models to humans has led to questions about the utility of *in vivo* studies and demand for more predictive models and tools based on the latest technologies. The current paradigm where animal models of allergen sensitization and challenge are considered the gold standard falls some way short of human asthma. New models need to be developed that take into account emerging knowledge underpinning the mechanistic basis of the disease and that reflect: (i) the chronic nature of asthma; (ii) environmental factors that drive asthma development and exacerbations; (iii) the importance of smooth muscle and other aspects of airway wall remodeling in asthma pathophysiology; and (iv) the role of airway epithelium in translating immunological and inflammatory reactions.

To encourage the asthma research community to rise to this challenge and move these aspirations forward the UK NC3Rs (National Centre for the Replacement, Refinement and Reduction of Animals in Research; http://www.nc3rs.org.uk) has initiated a programme of work to identify opportunities to develop scientifically and clinically more relevant models for basic and applied research [1]. This includes increased investment in the development of more complex human-based microfluidic *in vitro* models to study the dynamic interplay between static and mobile cells in the asthmatic airways; and to examine how environmental insults interact with asthma susceptibility genes [2]. The integration of data from human and *in vitro* studies is a key factor to meeting the criteria for new model development highlighted above, but will require greater cross-discipline and sector collaboration. In 2011 the NC3Rs worked with asthma researchers from the University of Nottingham to submit a challenge to the EPSRC-funded Mathematics in Medicine Study Group to explore the potential of mathematical modelling to better understand airway smooth muscle cell proliferation and apoptosis in asthma [3]. This partnership has identified some interesting gaps in knowledge in this area and has led to a number of follow-up activities between the asthma experimentalists and mathematicians. A subsequent NC3Rs Maths Study Group in 2013 also attracted a challenge from the asthma field with researchers from Imperial College London seeking mathematicians to work with on modelling steroid responsiveness in severe asthma and COPD [4]. To build on this early work the Centre has established an expert Asthma Advisory Group (AAG) which comprises researchers from across academia and industry. Chaired by Professor Stephen Holgate, the AAG advises the NC3Rs on further opportunities for advancing the 3Rs in basic asthma research and drug development. Initial activities of the Group will focus on the application of the 3Rs in severe asthma.

The future development of safe and efficacious new asthma therapies (and indeed therapies for other respiratory diseases) should be based on human data and knowledge of the human disease rather than the continued use of animal models which do not reflect the clinical disease. Adopting a more collaborative rather than competitive model is crucial to realising this.

## Asthma overview

Asthma is a chronic inflammatory disorder of the large airways that is associated with structural remodelling. A large number of infiltrating inflammatory cells are either activated or recruited into the asthmatic airway including dendritic cells, mast cells, macrophages, T-cells and eosinophils. In addition, resident structural cells including epithelial cells, smooth muscle cells are fibroblasts are activated and have abnormal cellular function in asthma [5,6]. Changes in cell activation are linked to enhanced expression of pro-inflammatory proteins including cytokines, chemokines, growth factors, neuropeptides, enzymes, receptors, and adhesion molecules and reduced expression of anti-inflammatory mediators such as IL-10 [7]. Overall, these changes result in the functional abnormalities associated with asthma such as variable airflow obstruction and bronchial hyper-responsiveness [5,6]. A large proportion of asthmatics are allergic and this has led to the concept of asthma as a Th2-driven eosinophilic disease of the airway. Indeed, most animal models of asthma utilize this concept for the development of airway hyper-responsiveness and inflammation [8].

Recent developments, however, are changing our views about the pathophysiology of asthma. Transcriptomic analysis of bronchial epithelial cells, for example, has confirmed the presence of distinct subsets of mild/moderate asthma and identified a gene profile– a high Th2 phenotype - that predicts a glucocorticosteroid (GC)-responsive asthma group of patients [9]. Importantly, this Th2 signature is present in only 50% of asthmatics indicating that the others do not fit the classic asthma paradigm. The Th2-high signature is linked to markers of airway remodelling [9], shows some variability [10] and may be a biomarker for patients who will respond to anti-IL-13 therapy [11]. There is increasing interest regarding the role of novel immune cells in asthma including Th17 and Treg cells and the release of specific mediators such as IL-17 which can induce steroid unresponsiveness in the mouse [12] and in primary human epithelial cells [13].

Furthermore, it is increasingly evident that asthma, and in particular severe asthma, is not a single disease state as evidenced by the variety of clinical presentations, physiologic characteristics and outcomes [14]. Detailed or deep phenotyping has developed in order to try to understand this heterogeneity better and is an important step towards improved understanding of this disease. Ultimately it is hoped that these asthma phenotypes will evolve into asthma ‘endotypes’, which combine clinical characteristics with identifiable mechanistic pathways [14].

Asthma phenotypes, to date, revolve around clinical characteristics rather than disease mechanisms. Unbiased hierarchical clustering of clinical characteristics [15,16] has identified several clusters of asthmatics. The adult Severe Asthma Research Program (SARP) cohort identified 5 clusters of asthma across the spectrum of mild, moderate and severe asthma. These included 3 groups of mild, moderate and severe early-onset atopic asthmatics, a more severe late-onset obese older women subgroup who frequently use oral corticosteroids, and a later onset but long duration very severe, less atopic group with less reversible airflow limitation [16]. Another adult asthma cohort analysis from Leicester included sputum eosinophil counts and identified 4 clusters including a similar early onset atopic-asthma, an obese non-eosinophilic-asthma, an early onset symptom predominant-asthma, and a later onset inflammation predominant asthma [15]. In both cluster analyses, severe asthmatics were distributed among several clusters supporting the heterogeneity of severe asthma. These unbiased phenotypes have substantial overlap with phenotypes previously recognized clinically (late onset eosinophilic and early onset atopic/allergic) [14].

The failure of inhaled or even oral glucocorticoids to suppress inflammation is seen in some patients with severe asthma [17]. These patients present a major healthcare problem and account for a large percentage of the overall costs for asthma worldwide [17]. These patients should not be confused with those who either do not take their anti-inflammatory medication or patients who do not have access to the correct treatments [17]. Currently, there is evidence that subsets of patients with severe treatment-insensitive inflammatory diseases including asthma will respond to specific anti-inflammatory agents e.g. anti-IL-5 [18], anti-IL-13 [11] and JAK/STAT inhibitors [19]. An alternative strategy is to attempt to restore the ability of steroids to suppress the inflammatory response in these patients which has been rendered defective [20].

Infection, whether bacterial or viral, causes asthma flares or exacerbations and is associated with a worse disease prognosis [21]. Infection causes enhanced airway inflammation, airway hyper-responsiveness and mucus hypersecretion possibly as a result of increased expression of inflammatory mediators and substances and greater accumulation of inflammatory cells in the airway mucosa and submucosa [21]. Both bacterial and viral infections may also affect glucocorticoid responsiveness [21].

The recent Models of Respiratory Disease Symposium presented several studies that investigated models of asthma. The classic models of allergen-induced asthma relate to short-term sensitization and exposure of mice to allergens such as ovalbumin (Ova) or house dust mite (HDM) as these produce a good Th2-mediated eosinophilia. Several abstracts investigated the mechanisms underlying HDM-mediated inflammatory responses.

A comparison between the anti-inflammatory effects of a corticosteroid and a phosphodiesterase (PDE) 4 inhibitor in the 5 week chronic HDM model was described (**P1, Jordan et al**). Chronic HDM extract exposure resulted in bronchoalveolar lavage (BAL), perivascular, peribronchiolar and alveolar inflammation which all increased in severity during the five week exposure period and was accompanied by epithelial and mucus cell hypertrophy/hyperplasia. Both roflumilast (10 mg/kg) and prednisolone (10 mg/kg), administered orally twice daily from Week 3, significantly inhibited BAL fluid cell recruitment and reduced the severity of the airway remodelling.

Comparisons between an acute and chronic HDM model were presented (**P2, Bunting et al**). A 10 day consecutive i.n. challenge with HDM elicited an early phase cytokine response as evidenced by significant increases in G-CSF, TNFα, IP-10 and KC in the lung on day 1 which could be linked to mechanisms driving inflammation such as neutrophil and macrophages/monocytes chemotaxis. The height of the inflammatory phase in the acute model is at day 7 with resolution seen by day 16. In contrast, in the 5 week chronic HDM model there is significant mucus overproduction and airway remodeling characteristics such as significant upregulation of soluble collagen and TIMP-1 which has been shown to be increased in sputum and lung biopsies from asthmatic patients.

Mice repeatedly challenged with HDM extract developed a robust airway neutrophilia rapidly evolving into asthma-like disease with increased numbers of BAL eosinophils and lymphocytes as well as inflammatory infiltrates, vascular/muscular hypertrophy, interstitial fibrosis, epithelial hyperplasia and mucus accumulation in lung tissues. RNA and protein screening revealed a robust Th17 component post-HDM exposure. Hubeau and colleagues (**P3, Hubeau et al**) were able to demonstrate that IL-17A deficient mice had reduced numbers of BAL macrophages, neutrophils, eosinophils and lymphocytes in response to chronic, but not acute, HDM challenge. Similar results were seen with anti-TL1A a member of the TNF superfamily known to promote Th2 and Th17 responses. However, the effects on lung function were minimal.

Imaging, has been neglected in animal models to a certain extent but a collaboration between GSK and AZ (**P4, Changani et al**) demonstrated the power of longitudinal imaging in the 7 week chronic HDM model. They were able to describe how MRI & CT methods provide sensitive early readouts of lung inflammation where individual animals can be tracked throughout a study enabling longitudinal intervention, potentially reducing animal numbers & providing a translational approach. Mice were scanned weekly using MRI or CT and AHR & IgE measurements were taken on weeks 3, 5 & 7. BAL & lungs were obtained following the last imaging session. MRI showed a gradual dose-dependent weekly increase in lung tissue intensity (LTI) in animals treated with HDM with respect to control. A corresponding increase in AHR, cell counts & IgE were observed. CT showed significant increases in LTI from week 1 of HDM & this was maintained throughout the 7 weeks. Budesonide treatment showed a reversal in the increase in LTI demonstrating that MRI & CT can provide a non-invasive & sensitive method for longitudinally assessing lung inflammation in this model.

The role of acidic mammalian chitinase was examined in short term Ova and HDM plus cockroach allergen models (**P5, Fitz et al**). Treatment with small molecule inhibitors of AMCase resulted in a significant reduction in allergen-induced chitinolytic activity in the airways but exerted no apparent effect on pulmonary inflammation. A lack of effect was also seen in transgenic and AMCase-deficient mice. However, in mice challenged with intratracheal HDM only without adjuvant, BAL neutrophil and lymphocyte counts were significantly increased in AMCase-deficient mice, whereas concentrations of IL-13 in BAL were significantly decreased compared with WT control mice. The authors conclude that although exposure to allergen stimulates the expression of AMCase and increased chitinolytic activity in murine airways, the overexpression or inhibition of AMCase exerts only a subtle impact on airway allergic disease. In addition, AMCase contributes to the Th1/Th2 balance in the lungs which may be of importance in asthmatics with enhanced airway neutrophilia.

Whilst extended HDM protocols are useful for investigating features of lung remodelling, the allergic phenotype, consisting of the cell and cytokine/chemokine network, usually develops earlier. Using a 3 week (5 days per week) challenge model Uddin and co-workers (**P6, Uddin et al**) demonstrated that all BAL cell types bar macrophages were elevated above background levels 4 hours after the last HDM challenge and this correlated with increased chemokine expression. Serum total IgE levels were also elevated with a small, above background increase in HDM-specific IgE. HDM challenged animals exhibited an increased AHR compared to saline challenged animals. The modified protocol allows for the investigation of allergic mechanisms at an earlier time-point resulting in less cost to animals and a quicker data turnaround time.

Due to the limitations of the chronic HDM model [1], new models are being developed and several were discussed at the meeting. In one model, airway inflammation and AHR were elicited by s.c. sensitisation to HDM in complete Freund’s adjuvant (CFA) and a subsequent i.n. HDM challenge into the airways 14 days later (**P7, Dixon et al**). The resulting inflammation consisted of a combined eosinophilia, neutrophilia and a mixed Th1, Th2 and Th17 cell response. IFNγ neutralisation profoundly inhibited AHR yet enhanced BAL eosinophilia, neutrophilia, IL-17 levels and HDM specific serum IgE. Conversely, anti-IL-4 treatment inhibited eosinophil accumulation, IL-13 production and HDM specific serum IgE and IgG1, without affecting AHR. Anti-IL-17 treatment reduced BAL neutrophilia and KC levels, however no significant modulation of AHR, eosinophilia or HDM specific serum IgE and IgG1 was observed. The data indicates that this model has a complex immunology similar to that seen in some subjects with severe asthma and is relatively steroid refractory. Importantly, there is a dissociation between cellular inflammation and hyperreactivity of the airways in this model which is similar to that seen in severe asthmatics.

A number of rat allergen-induced models were presented. The most objective indicator of asthma severity in the clinic is the measurement of reversible airway obstruction by spirometry. Jordan and colleagues (**P8, Jordan et al**) evaluated the effect of antigen challenge on FEV_100_, FVC and PEF and correlated these with airway inflammation in the Brown Norway rat Ova model. Ova challenge caused a significant reduction in FEV_100_, PEF and FVC accompanied by a significant recruitment of eosinophils, neutrophils and lymphocytes into the airway. Furthermore, IL-13 and IL-5 and macrophage derived TNF-α in addition to IL-6 and MIP-1α levels were significantly elevated in the BALF. These changes were all reversed by budesonide (3 mg/kg) twice daily.

Similar data was presented by Woodhouse and co-workers (**P9, Woodhouse et al**) who demonstrated that Betamethasone inhibited BAL neutrophilia and inflammatory mediator expression in a dose-dependent manner at time points ranging from 6-48h.

*Alternaria alternata* is a fungal allergen linked to the development of severe asthma and is able to elicit a robust immune response in the lungs. The effects of a single intratracheal (i.t.) instillation of *Alternaria* on immune responses in the Brown Norway rat was investigated (**P10, Gil et al**). *Alternaria* exposure produced a dose- and time-dependent recruitment of neutrophils in the lungs along with increased and cytokine levels in BALF. This was associated with enhanced activation of the JAK/STAT pathway. Dexamethasone administered prior to *Alternaria* instillation led to a dose-dependent attenuation of *Alternaria* induced airway inflammation. This preliminary profiling suggests that *Alternaria* challenge has the potential to be a robust and reliable PK/PD model to assess *in vivo* compound potency.

Understanding the role of the mast cell in asthma has proved difficult due to a lack of effective mast cell-directed agents (**P11, Crackower et al**). Spleen tyrosine kinase (SYK) is a key activator of signaling pathways downstream of the high-affinity IgE receptor (FcεR1) in mast cells. A selective SYK inhibitor (MRK-A) dose-dependently blocked IgE-mediated tracheal extravasation in Ova-challenged rats and dose-dependently inhibited airway inflammation via the oral route. In addition, i.v. MRK-A significantly inhibit both the early and late allergen-induced changes in airway resistance and AHR in an ascaris-sensitive sheep allergen challenge model. These models implicate a key role for this pathway in allergic airways disease.

Uddin and colleagues (**P12, Uddin et al**) further characterised the Werner-Klein model of Ova-induced rapid pulmonary inflammation that exhibits rapid and long lasting pulmonary eosinophilic infiltration requiring only a short sensitisation period. Male Brown Norway rats, 7-9 weeks old, were sensitised to OVA (200µg) emulsified in aluminium hydroxide (2.66mg) via the intraperitoneal route on days 0, 1 and 2. On days 5 and 6, rats were challenged with aerosolised 1% OVA and the bronchoconstrictor response after challenge on day 6 was measured. Significant levels of all cell types were present in the BAL from day 7 and eosinophil and CD4+ lymphocytes remained high up to day 15. OVA-induced bronchoconstriction was apparent only after the re-challenge at day 15 while at day 6 it was indistinguishable from saline challenged animals. The speed and robustness of the model has implications with respect to the 3R’s impact but further characterisation is required.

Timing of drug administration may be particularly important and some evidence was presented indicating that this is true in animal models of asthma. Circadian oscillations of lung mechanical properties have been reported in conscious undisturbed rodents. Potentially, these fluctuations could alter the pulmonary response induced by allergen provocation and the response of pharmacological treatments in asthmatics. In a very interesting study, Otal and colleagues (**P13, Otal et al**) demonstrated that there was no difference in the severity of the LAR in rats whether evoked in the morning, afternoon or evening but that they were more frequent in the evening. In contrast, the LAR triggered in mice in the morning was more severe and more frequent than that evoked during the evening. These results suggest that species and day period of time to induce LAR in allergic animal models are critical and should be taken into account when evaluating the effects of new compounds.

Breeding of beagles over 20 years at the Lovelace clinic has resulted in animals that need ever higher dose of steroids to see a similar anti-inflammatory effect (**P40, Barrett et al**). In these studies, dogs (n=6-8/study) previously sensitized to ragweed (RW) allergen were pretreated with vehicle or a) 1 week oral prednisolone (15 mg/day), b) 2 weeks oral prednisolone (15 mg/day), or c) 2 weeks inhaled fluticasone propionate (350 µg lung deposited dose; BID). One week of prednisolone treatment did not lead to any significant attenuation of pulmonary function endpoints or lung inflammation whereas 2 weeks of prednisolone treatment led to a significant reduction in BAL cells (neutrophils, eosinophils, and lymphocytes) and in blood eosinophils but did not affect airway function. Similarly, 2 weeks of inhaled fluticasone led to a significant reduction in BAL cells and blood eosinophils without affecting airway function. These dogs exhibit some features of a severe steroid-resistant phenotype which may be useful in further studies in this area.

Toll-like receptors are important in driven innate immune responses including those involved in asthma. Initial studies in ragweed (RW)-sensitized dogs, demonstrated that the selective TLR8 agonist VTX-378 improved nasal congestion in a dose-dependent manner (**P14, Barrett et al).** In a subsequent human clinical study, VTX-1463 demonstrated significant improvements in Total Nasal Symptom Score (TNSS, sum of scores for nasal congestion, itching, sneezing and rhinorrhea) in grass pollen allergic subjects in season. There was also a trend towards a benefit in Active Anterior Rhinometry (ARR) but this did not reach significance. This suggests that in the context of nasal allergies the dog model can be predictive of dose and clinical efficacy.

The ascaris-sensitive sheep model has many features of human asthma including an early airway response (EAR), a late airway response (LAR) and airway hyperreactivity (AHR) (**P15, Caniga et al**). As expected, corticosteroids show little inhibition of the EAR at low doses but completely inhibit the LAR and AHR. Interestingly, the chemoattractant receptor-homologous molecule expressed on T-helper type 2 cells (CRTH2) inhibitor, MK-7246, had a similar profile to corticosteroids with little inhibition of the EAR and blockade of the LAR and AHR. Since current gold standard drugs including corticosteroids and β_2_-agonists perform similarly in this model as in man, this model may offer some key element of airways reactivity function similar to human asthma. However, further translational work is required to confirm this.

Some human and non-human primate data was also presented. Cynomolgus monkeys (*Macaca fascicularis*) are naturally sensitized to *Ascaris suum* antigen in the wild and develop a reproducible model of acute airway inflammation following segmental *A.suum* antigen challenge **(P16, Bree et al**). The inflammation was characterized by an increase in total BAL cell counts and increased eosinophils associated with a trend towards increased IL-5 and eotaxin all of which were dexamethasone sensitive. This model should be useful for testing the efficacy of selected anti-inflammatory drug candidates.

There are differences between the human and mouse immune systems. To obviate this, Schaumann and colleagues used a human *in vitro* allergy model to provide an alternative method to investigate allergy driven immune responses (**P17, Schaumann et al**). Antigen-presenting cells (APCs) were generated from CD14+ monocytes obtained from whole blood. APCs were stimulated with HDM before cells were harvested and co-cultured with autologous CD4+ lymphocytes. Both, the generated and HDM-pulsed APC induced strong proliferation of the co-cultured CD4+ lymphocytes which was inhibited by the presence of plasmacytoid dendritic cells (pDC). Similar effects were seen in HDM-stimulated PBMC. This model mimics allergen-specific immune responses and has the advantage of possessing human immune regulatory mechanisms. In the future, this model might be useful for testing the applicability of potential immunoregulatory drugs.

The early allergic reaction (EAR) is not present in mouse models of asthma despite being an important feature of the human disease. OVA-sensitized guinea pigs, in contrast, have a good EAR. Riley and colleagues (**P18, Riley et al**) used a combination of histamine 1 receptor, cysteinyl leukotriene 1 receptor, and cyclooxygenase inhibitors alone or in combination to examine the contribution of mast cell mediators to the EAR and airway hyper-responsiveness. This highlights the potential of the guinea pig as a model of the EAR.

Respiratory viral infections are commonly associated with the exacerbation of asthma in humans and more predictive animal models of asthma exacerbation are needed [5,6]. Human rhinoviruses (HRV) cause the majority of common colds and acute exacerbations of asthma and effective therapies are needed since no licensed treatments or vaccines currently exist [5]. Corticosteroids are frontline treatment in asthma. Individuals who are corticosteroid insensitive suffer from frequent exacerbations of clinical symptoms, which further decrease corticosteroid sensitivity [7].

Shaw and colleagues (**P19, Shaw et al**) presented a large body of work examining the ability of HRV (both RV16 and RV1b) to induce an inflammatory response and exacerbate the chronic HDM model. They were unable to detect either HRV1B or HRV16 replication in the lungs and only a variable inflammatory response up to 24 hours post inoculation. In contrast, animals treated with influenza A/Victoria/3/75 H3N2 strain readily replicated in the lungs and developed a mixed inflammatory lung inflammation. H3N2 was also shown to replicate very well in the mouse. There was no indication that a single inoculation of HRV1B superimposed upon the chronic HDM phenotype altered the HDM-induced AHR or inflammatory profile. In comparison, the combination of H3N2 with chronic HDM treatment resulted in an increase in BAL eosinophils and neutrophils. No change in steroid responsiveness was observed.

In BALB/c mice sensitized intranasally with house dust mite (HDM) extract (25µg in 50µl saline) for five days per week over 7 weeks, intranasal HRV1b was unable to induce an exacerbation phenotype as determined by a lack of effect on AHR, BAL inflammatory cell counts, draining lymph node cell counts and *ex vivo* proliferative response (**P20, Rochlitzer et al.**). In contrast, in naïve mice, HRV1b infection resulted in an impaired anti-viral immune response represented by reduced BALF neutrophil cell counts, cytokine levels as well as *ex vivo* proliferative response of draining lymph node cells. This data suggests that although the models may not exacerbate, the presence of an impaired anti-viral response might have functional consequences.

The failure of HRV to induce an exacerbation in the chronic HDM model might be due to inefficient viral replication or the fact that the immune response in these HDM-treated animals is too skewed. Other HDM models have been developed that appear to have a mixed lymphocyte component and are relatively steroid insensitive (**P21, De Alba et al**). In this model, BALB/c mice were sensitised subcutaneously on day 0 with HDM (100µg) in -CFA and on day 14, mice were exposed to saline or HDM (25µg) via intranasal instillation. Poly I:C (30 µg) was administered at various time points before or after the HDM challenge. Poly I:C exacerbated BALF neutrophils, macrophages and lymphocytes in the HDM challenged animals which was accompanied by an increase in AHR.

Other studies presented at the meeting demonstrated a clear exacerbation of the chronic HDM model using other viruses. For example, Barrett and colleagues using the chronic 5 week HDM model determined the effect of prophylactic fluticasone treatment on subsequent infection by influenza (A/HKx31[H3N2] strain, 10^4^ PFU) (**P41, Barrett et al**). The effect of chronic HDM exposure on BAL inflammation and AHR was reversed by fluticasone. Although the addition of the A/HKx31[H3N2] influenza strain did not induce an exacerbation phenotype as measured by inflammatory cell counts and AHR, the viral infection reduced the efficacy of fluticasone to resolve the lung inflammation and AHR induced by HDM resulting in elevated BAL cells and AHR, both features of an exacerbation.

Similarly, another group (**P22, Lamb et al**) also reported on the ability of RSV and influenza to exacerbate the chronic HDM model and the ability of fluticasone to impact upon some of these readouts. Groups of mice were sensitised and challenged in the 5 week chronic HDM model and then infected with 5 x virus doses (either RSV or influenza A) and virus replication and inflammatory responses monitored at days 1, 3, 7 and 14 post infection. Overall both viruses were able to exacerbate some aspects of the chronic HDM-induced inflammatory response and some of these responses became relatively steroid insensitive. In detail, RSV increased HDM-induced BAL neutrophilia and KC expression was steroid sensitive whilst the enhanced BAL lymphocyte numbers and exhaled nitric oxide levels were only partially steroid sensitive. In contrast, RSV inhibited HDM-elicited eosinophilia. Influenza A elicited increases in BAL neutrophils and lymphocytes independently of HDM challenge were steroid insensitive as was the influenza/HDM-induced increase in BAL lymphocytes. Influenza A inhibited HDM-elicited eosinophilia and modestly increased exhaled nitric oxide levels. The latter was steroid insensitive. Importantly, influenza A increased 5-HT-mediated AHR which was steroid insensitive and although not synergising with the enhanced AHR seen with HDM-treatment this became steroid sensitive.

There are differences in the responses seen with influenza strains which may reflect the virulence of the strains or the precise model used **(P23, Bal et al)**. As such, in an acute asthma HDM model (sensitised intranasally with HDM on three consecutive days and challenged two weeks later) there was a significantly higher influx of eosinophils into the lungs of the HDM-treated mice compared to the HDM/PBS and the PBS/influenza group. Furthermore, more IL-5 was produced on day 4 after influenza infection (10 TCID_50_ influenza A/PR/8/34 strain) in the lungs of HDM-treated mice compared to PBS/influenza-infected mice, whereas the IFN-γ production on day 8 was decreased. There was a trend of elevated AHR on day 3 after infection. In contrast, there was a significant exaggeration of AHR in the chronic HDM following influenza linked to increases in BAL neutrophils and eosinophils.

In a subsequent study the same group examined the role of the eosinophil in the influenza-induced exacerbation (**P24, Bal et al**). IL-5 transgenic mice, which present with chronic eosinophilia, were infected with 10 TCID_50_ influenza (A/PR/8/34) and demonstrated a peak BAL eosinophilia at day 4 that was >10-fold greater than in wild type animals. The IL-7 Tg mice recovered much earlier from infection than wild type animals, had a much reduced weight loss and a lower viral load. This data indicates that in mice at least, IL-5 and eosinophils play a crucial role in the immune response against influenza virus in mice. This response is difficult to reconcile with clinical data in asthmatic patients treated with anti-IL-5 antibody, who have been shown to suffer significantly fewer (mostly virally-induced) exacerbations; further work is needed to understand this.

The meeting finished with workshops devoted to specific topics and the major areas of discussion are considered below:

## Exacerbation Workshop session

Despite current standards of care, acute exacerbations due to viral-induced inflammation remain a major cause of asthma morbidity, mortality and health-care costs. One symposium session focused on pre-clinical animal models of human viral exacerbations followed by a roundtable workshop (**P19-P24, P41**). The RV16 challenge model is one clinical exacerbation model that was described in the session’s plenary talk given by Prof. Sebastian Johnston. During the workshop, we discussed the need to obtain a more complete profile of the human RV16 challenge model and naturally exacerbating patients in order to determine which mechanisms and pathways are represented in specific animal models currently in use or under development.

A collaborative effort between academia and industry to address this need was highlighted by several presentations from the U-BIOPRED consortium. Alba et al. (**P21)** described the CFA/HDM model, and highlighted the use of this mixed Th1/Th2/Th17 inflammation model to study an exacerbation triggered by poly I:C. The eventual goal is to compare the pathways active in this model to the profile generated from a human RV16 challenge study that is part of the U-BIOPRED clinical study. This presentation triggered a lively workshop discussion regarding the use of virus versus synthetic analogues such as poly I:C. The consensus was that poly I:C would continue to be used since not all institutions have the infrastructure to conduct viral studies. As in the case of complex antigens such as HDM, the group also shared experiences with variability in study results being attributed to the source or lot of poly I:C, and the need for high titer, viral stocks.

As previously discussed above, two presentations highlighted the protective role of eosinophils in a murine model using an influenza exacerbation (**P24 and P41**). These studies led to a discussion about the translatability of murine exacerbation models given the recent clinical data that anti-IL5 reduces exacerbations. In addition, workshop discussion focused on the need to fully characterize animal models using a wide variety of endpoints. However, in the context of current translatable endpoints, airway inflammation and lung function remain the primary focus with considerable interest in pursuing new imaging techniques that enable longitudinal sampling of a single cohort of animals.

Workshop discussion also focused on the difficulty of exacerbating standard lung inflammation models. Both Rochlitzer et al. (**P20**) and Barrett et al. (**P41**) used the chronic HDM model but were unable to observe an exacerbated phenotype with HRV1b or influenza, respectively. One significant challenge is to develop a reproducible model of underlying airway inflammation that also provides a window to measure an exacerbation. In addition, this exacerbation window would have to enable pharmacological testing of new mechanisms of interest or current standards of care.

Finally, workshop participants also stressed the importance of publishing negative data associated with model development or pharmacological characterization of an established model. A quarterly themed issue published by an established journal was one suggestion to address this need and continue the open sharing of knowledge between academia and industry.

## Asthma Workshop Session

This workshop within the Symposium focussed on issues of the species used to model asthma in humans, and participants from several academic and company groups contributed.

Whilst mice are clearly extensively used for reasons of convenience in husbandry and handling, the availability of reagents, ethical considerations, and the unique degree of availability of transgenic and knock-out mice, they have a long list of drawbacks. These include significant differences in the anatomy and pharmacology of the lung, the absence of cough, the difficulty of measuring lung function in a way that reflects human FEV_1_ etc measurements, the difficulty in seeing early and late phases of response to allergen challenge. It is also the experience of workshop participants that studies of inhaled drug delivery are difficult to carry out and to extrapolate to inhaled dosing of drugs to humans.

A species that has been used historically for asthma research and which addresses some of these drawbacks is the guinea pig (GP) (**P18 Riley et al**). These are much more amenable to investigations of lung function, have a lung anatomy and pharmacology (e.g. beta 2 adrenergic receptor distribution and function) more similar to human and can be used in studies of cough. However there are significant limitations to using GPs to model human asthma: inconsistent/ drifting responses to allergen over time and between laboratories, and the paucity of reagents such as GP cytokines and chemokines and antibodies for the quantification or neutralisation of these.

Other species have been frequently used to model asthma, albeit to a more limited extent: rats, dogs, sheep and non-human primates (marmoset, rhesus and cynomolgus monkeys). Rats and dogs have some particular advantages, e.g. in being standard safety evaluation species, such that pharmacological activity can be related to safety thresholds. Moreover, delivery of compounds by inhaled routes and extrapolation of doses to clinical use is more straightforward and precedented in these species. Non-human primates have the advantage of being more closely homologous to humans at the gene sequence level, although this comes with ethical and animal handling considerations that have to be taken into account.

The issue of the lack of reagents for research in GPs is one that the workshop participants considered potentially addressable, and it was agreed that a follow-up working group would be organised to look into this, with particular involvement of Karolinska (Adner), University of Cardiff (Ford) and Imperial College (Belvisi & Adcock) as well as some of the particularly interested companies.

## COPD Workshop Session

COPD is a complex inflammatory airway disease that results in airflow limitation that is not fully reversible. Many animal model systems have been developed that recapitulate various features of COPD but all still suffer from significant limitations (**P25, Vlahos**). The lack of understanding of the underlying mechanisms and mediators that drive the onset and progression of chronic inflammation, emphysema and changes in lung function have limited the development of useful models and subsequently effective treatments. This Symposium session and workshop focused on identifying current preclinical animal modeling systems that are being utilized to evaluate new therapeutics targeted for treatment of COPD and identify their strengths and shortcomings.

Many of the presentations highlighted different flavors of the common models that are actively being utilized ranging from acute (< 14 days) ozone (**P27 Armstrong et al**), smoke (**P26 Schlerman et al, P27 Armstrong et al, P30 Ramaprakash et al, P31 Russell et al, P32 Russell et al) and LPS (P28 Seehase et al, P29 Schlerman et al**) exposure to more sub-chronic versions of each (**P25, Vlahos**). Both the smoke and ozone models consistently show a refractory response to steroids while the LPS challenge models are more responsive. While it was agreed that the chronic models show more robust lung pathology (e.g. development of emphysematous changes) there were practical concerns raised in terms of the duration and cost of these models in the preclinical drug development setting. The acute models were also highlighted with respect to their potential utility in overlaying a viral challenge to induce an acute exacerbation (**P32, Russell et al, P33, Kubera, P34, Schlerman et al**), that in combination, leads to lung changes more similar to the chronic exposures, but in a much shorter time. Interestingly, there seemed to be a divergence between the COPD and asthma models with respect to their duration as there was a broad effort to shorten the COPD models and shift to longer allergen challenge models for asthma (due to the lack of therapeutic predictability with acute challenges).

While most of the experimental designs relied on the use of rodents there were two presentations which highlighted the use of non-human primates (common marmoset and cynomolgus) in conjunction with LPS challenge (**P28 Seehase et al, P29 Schlerman et al**). It was felt that while there might be some specialized use for these models depending on the pre-clinical target being developed these types of models would not likely become widely used as their responses to standard therapies (e.g. steroids and PDE4 inhibitors) seemed similar to rodent models and their expense could be prohibitive.

While all felt the challenges were many in developing more useful animal models of COPD there was broad consensus that greater understanding from the clinic would ultimately be needed in order to drive the development of models that recapitulate more features of the disease. There was enthusiasm to continue these types of discussions between industry and academia and clinicians and bench scientists to further develop appropriate models to aid preclinical drug development.

## Conclusions

Very positive unsolicited feedback was received from many meeting participants. The symposium was considered to be a valuable meeting, in an unusual forum with the opportunity for significant interchange between companies and companies with academia. It was felt that the environment created was conducive to the exchange of information regarding challenges and issues as well as collaborative and open debate. A key strength of the meeting was that negative as well as positive data was openly shared in order to try and avoid unnecessary duplication of animal studies.

Despite the challenges we face as a community of respiratory biologists, participants from both academia and industry were enthusiastic about finding future opportunities to interact and discuss progress in a collaborative manner. Workshop participants were interested in evening seminars on this topic during ERS or ATS in addition to highly focused meetings such as this one. Indeed the organizing committee is considering another Respiratory Symposium in May 2014, potentially to coincide with the American Thoracic Society Conference in San Diego, U.S.A.
